# Zinc-Induced SUMOylation of Dynamin-Related Protein 1 Protects the Heart against Ischemia-Reperfusion Injury

**DOI:** 10.1155/2019/1232146

**Published:** 2019-07-22

**Authors:** Xiyun Bian, Jingman Xu, Huanhuan Zhao, Quan Zheng, Xiaolin Xiao, Xiaofang Ma, Yanxia Li, Xinping Du, Xiaozhi Liu

**Affiliations:** ^1^Central Laboratory, The Fifth Central Hospital of Tianjin, Tianjin 300450, China; ^2^Medical Research Center, North China University of Science and Technology, Tangshan 063000, China; ^3^Department of Physiology and Pathophysiology, Tianjin Medical University, 300070 Tianjin, China; ^4^Department of Cardiology, The Fifth Central Hospital of Tianjin, Tianjin 300450, China

## Abstract

**Background:**

Zinc plays a role in mitophagy and protects cardiomyocytes from ischemia/reperfusion injury. This study is aimed at investigating whether SUMOylation of Drp1 is involved in the protection of zinc ion on cardiac I/R injury.

**Methods:**

Mouse hearts were subjected to 30 minutes of regional ischemia followed by 2 hours of reperfusion (ischemia/reoxygenation (I/R)). Infarct size and apoptosis were assessed. HL-1 cells were subjected to 24 hours of hypoxia and 6 hours of reoxygenation (hypoxia/reoxygenation (H/R)). Zinc was given 5 min before reperfusion for 30 min. SENP2 overexpression plasmid (Flag-SENP2), Drp1 mutation plasmid (Myc-Drp1 4KR), and SUMO1 siRNA were transfected into HL-1 cells for 48 h before hypoxia. Effects of zinc on SUMO family members were analyzed by Western blotting. SUMOylation of Drp1, apoptosis and the collapse of mitochondrial membrane potential (ΔΨm), and mitophagy were evaluated.

**Results:**

Compared with the control, SUMO1 modification level of proteins in the H/R decreased, while this effect was reversed by zinc. In the setting of H/R, zinc attenuated myocardial apoptosis, which was reversed by SUMO1 siRNA. Similar effects were observed in SUMO1 KO mice exposed to H/R. In addition, the dynamin-related protein 1 (Drp1) is a target protein of SUMO1. The SUMOylation of Drp1 induced by zinc regulated mitophagy and contributed to the protective effect of zinc on H/R injury.

**Conclusions:**

SUMOylation of Drp1 played an essential role in zinc-induced cardio protection against I/R injury. Our findings provide a promising therapeutic approach for acute myocardial I/R injury.

## 1. Introduction

Myocardial ischemia-reperfusion (I/R) injury causes a variety of serious consequences, including ventricular fibrillation, heart rupture, and sudden death. Currently, there are few effective interventions to protect the heart against ischemia-reperfusion injury [1]. Sheng et al. [2] found that levels of zinc decreased in cardiomyocytes during reperfusion and zinc ion is one of the essential trace elements for the body. Zinc was involved in the regulation of more than 100 proteases, structural stability of cell membranes and organelles, and regulation of signaling pathways in various pathophysiological processes [3]. Moreover, the levels of various zinc transporters maintain zinc homeostasis during reoxygenation. Protein levels of ZnT1, ZnT2, ZnT5, and ZnT9 decreased, and protein levels of Zip2, Zip7, Zip13, and Zip14 increased [4]. These indicated that endogenous zinc ions played an important role in myocardial ischemia-reperfusion injury. Similarly, isolated rat hearts treated with exogenous zinc ions during reperfusion reduced the infarct size of the heart through some kinase pathways, and rat cardiomyocytes H9c2 treated with zinc ions during reoxygenation also reduced myocardial cell damage [5]. It is indicated that exogenous zinc ions also protect the myocardium from I/R or H/R damage. However, the exact protection mechanism of zinc ions needs to be further explored.

In the past ten years, a number of studies have shown that SUMOylation is involved in determining the fate of perfused heart [6, 7]. Currently, there are five mammalian SUMO paralogues (SUMO1, SUMO2, SUMO3, SUMO4, and SUMO5). The primary structural homology of SUMO1, SUMO2, and SUMO3 proteins is nearly 50%, and the homology of SUMO2 and SUMO3 proteins is about 97%. The structure of SUMO4 and SUMO5 is different from the other three SUMO proteins, and they have not been widely observed in mammalians [8, 9]. SUMO4, lacking of C-terminal processing, results in its inability to conjugate to lysine residues in target proteins [10]. SUMOylation is a dynamic reversible process and can be mediated by the SENP family. There are seven mammalian SENPs, including SENP1, SENP2, SENP3, SENP5, SENP6, SENP7, and SENP8. Of these, SENP8 shows a specificity against ubiquitin-like Nedd8 protein and does not reverse SUMOylation. Other SENPs have a different specificity for SUMOs. SENP1 and SENP2 have a broad specificity for SUMO1 and SUMO2/3, while SENP3 and SENP5 favour the removal of SUMO2, and SENP6 and SENP7 have less effect on SUMO2/3 monomer than poly-SUMO of SUMO2/3 [11]. The SUMO conjugation pathway is important for the development of a wide variety of human diseases such as brain ischemia and tumorigenesis [12–14]. Previous work also indicated that SUMOs targeting the proteins contribute to a number of human cardiovascular disease, such as valvular abnormalities, ischemic heart disease, cardiac hypertrophy, and idiopathic cardiomyopathy [15]. In animals subjected to heart I/R, SUMO1 conjugations were shown to be inactivated [16]. However, it is unclear, under these conditions, whether and how SUMO modification is involved in the protection of zinc ions against cardiac I/R injury.

Dynamin-related protein (Drp) 1 is a key protein for mitochondrial fission. It consists of four parts: GTP-binding, middle, insert B, and C-terminal GTPase effector (GED) domains. A variety of posttranscriptional modifications contribute to the regulation of Drp1 activity, such as phosphorylation, ubiquitination, SUMOylation, and S-nitrosylation, and SUMOylation appears to exert a role in the regulation of Drp1 activity [17, 18]. Studies have reported the removal of SUMO2/3 from Drp1 mediated by SENP3 and SENP5 and the removal of SUMO1 from Drp1 through SENP2 [6, 19, 20]. However, there is no evidence indicating a direct involvement of SUMOylation of Drp1 in the protection of zinc against cardiac I/R injury.

The aim of our study is to determine whether (1) the SUMOylation of Drp1 contributes to the progression of myocardial I/R injury and how it contributes to the protection of zinc preconditioning and (2) zinc preconditioning can induce mitophagy via regulating Drp1 SUMOylation in the heart.

## 2. Methods

This study conforms to the NIH Guide for the Care and Use of Laboratory Animals (NIH publication No. 85–23, revised 1996).

### 2.1. Chemicals and SUMO1 KO Mice

ZnCl_2_ and N,N,N′,N′- tetrakis-(2-pyridylmethyl) ethylenediamine (TPEN) was purchased from Sigma (St. Louis, MO, USA). SUMO1 KO animals were graciously made available by Professor Wei Yang and Huaxin Sheng (Duke University, Durham, North Carolina, USA) [7]. Wild-type C57Bl/6J mice were purchased from the Military Medical Science Academy Laboratory, Beijing, China.

### 2.2. Perfusion of Isolated Rat Hearts

Mice (*n* = 6/group) were anesthetized with isoflurane (300 mg/kg, intraperitoneal). After the mice were anesthetized, the PE-90 cannula with internal and external core structure was used for tracheal intubation. Inserted into the upper part of the tracheal bifurcation, pull out the inner core, intubate the ventilator, adjust the tidal volume of the mouse to maintain 250-300 ml/min, and adjust the respiratory rate to 110-130 times/min. The body temperature was maintained at 36.5-37°C using an infrared body temperature heater. The mice were fixed in the right lateral position, and a transverse incision was made from the left axilla. Open the thoracic cavity to expose the left ventricle and the left atrial appendage from the fourth or fifth intercostal space. Using the 7-0 lossless suture from the lower edge of the left atrial appendage about 2 mm through the myocardium surface to the side of the pulmonary artery cone, the ends of the suture were passed through a small piece of soft vinyl tubing to form a snare. Regional ischemia was induced by fixing the snare to the heart by clamping a hemostat. After 30 min of ischemia, the hearts were reperfused for 120 min by releasing the hemostat. Zinc (100 *μ*g/kg) was given 5 min before reperfusion for 30 min.

### 2.3. Measurement of IS

After 2 h of I/R, the left anterior descending artery was ligated, and 1 ml of 2% Evans blue dye (Sigma-Aldrich, St. Louis, MO) was injected from the abdominal aorta. After the heart turned blue, the perfusion was stopped and the heart washed thoroughly with normal saline to distinguish between nonrisk areas and area at risk (AAR). Remove the heart and store in a refrigerator at -20°C for 2 h. After the heart is frozen and fixed, cut a slice of about 1 mm from the apex, a total of 4 pieces. The heart was incubated in 1% triphenyltetrazolium chloride (TTC, Sigma-Aldrich, St. Louis, MO) at 37°C for 20 minutes to distinguish the infarct area (white, IS) from the risk area (red). The sections were fixed in 10% paraformaldehyde for 2 h to distinguish the stained areas from the unstained areas. The area of nonrisk areas, area at risk, and infarcted areas was analyzed by ImageJ software (National Institutes of Health, Bethesda, MD). The percentage of infarct size was calculated as IS/AAR × 100%.

### 2.4. Hypoxia/Reoxygenation of Cardiac Cells

Mouse atrial myocyte cell line HL-1 was obtained from the American Type Culture Collection (ATCC, Manassas, VA) and cultured in Claycomb Medium (Sigma, San Francisco, USA) with 10% fetal bovine serum (FBS) (Invitrogen, Carlsbad, CA) and 1‰ streptomycin at 37°C in the 5% CO_2_-95% air atmosphere. After cells grow to a density of 90%, digest with 0.25 mM trypsin, dilute to different densities, and inoculate them in different well plates, such as 6-well plates (5.6 × 10^4^ cells/well), 96-well plates (2 × 10^3^ cells/well), and confocal plates (2 × 10^4^ cells/plate). For hypoxia/reoxygenation (H/R) experiments, cells were cultured in low-glucose DMEM (Invitrogen, Carlsbad, CA) with free FBS and were placed in a hypoxic chamber (Coylab, Grass Lake, MI) and cultured with 95% N_2_/5% CO_2_ at 37°C for 24 h. During 6 h of reoxygenation, HL-1 cells were incubated in DMEM containing 10% FBS at 37°C in the 5% CO_2_-95% air atmosphere.

### 2.5. Plasmid Construction, siRNA Synthesis, and Transient Transfection into HL-1 Cells

Full-length cDNAs for mouse SUMO1, UBC9, SENP2, and Drp1 were obtained by RT-PCR using total RNA extracted from HL-1 cells, and their sequences were confirmed by BigDye sequencing (Applied Biosystems). SUMO1, UBC9, SENP2, and Drp1 full-length cDNAs were subcloned into the mammalian expression plasmid pcDNA3.1 (Life Technologies) harboring a HA, Flag, Myc tag at the C-terminus, respectively. Lysines 532, 535, 558, and 568 of Drp1 were replaced with leucine (Myc-Drp1 4KR) using the site-directed gene mutagenesis kit (Beyotime). siRNA oligonucleotides were synthesized by Sigma. The sequences were as follows: negative control siRNA: 5′-GAT CCG AAT TGC CAC AAC AGG GTC GTG TTC AAG AGA ATCA CAT CTT CTT CCT CCA TTC TTT TTTG-3′; SUMO1 siRNA: 5′-GAT CCG CCT TCA TAT TAC CCT CTC CTT TCA AGA GAA GGA GAG GGT AAT ATG AAG GCT TTT TTG-3′. The empty vectors, the target plasmid, negative control siRNA, and SUMO1 siRNA were transiently transfected into HL-1 cells using lipofectamine 2000 reagent (Invitrogen, Carlsbad, CA) according to the manufacturer's instructions. All experiments were conducted for 48 h after transfection.

### 2.6. Confocal Imaging of Mitochondrial Membrane Potential

The mitochondrial membrane potential (ΔΨm) was measured by JC-10 (Solarbio, Beijing, China), the mitochondrial membrane fluorescent dye. HL-1 cells were incubated with JC-10 according to the manufacturer's instructions. The fluorescence changes were detected with a laser scanning confocal microscope. The maximum excitation wavelength of JC-10 monomer is 515 nm and the maximum emission wavelength is 529 nm (green); the maximum excitation wavelength of JC-10 polymer is 585 nm, and the maximum emission wavelength is 590 nm (red).

### 2.7. Measurement of Reactive Oxygen Species

HL-1 cells were stained with 2,7-dichlorodihydrofluorescein diacetate (DCFH-DA,10 *μ*M/l) (Solarbio, Beijing, China) for 20 minutes in Claycomb Medium with free FBS. The cells were washed three times with serum-free medium and were directly observed by confocal microscopy with serum-free cultures, using 488 excitation light and 525 emission light to capture green fluorescence.

### 2.8. Western Blotting

After protein sample extraction, the concentration was consistently applied in an equal volume of 30 *μ*g and electrophoresed on 10-12% polyacrylamide gel. The protein was transferred to polyvinylidene fluoride membranes, blocked with 5% skim milk for 90 min, and incubated with the primary antibody at 4°C overnight. Primary antibodies included anti-SUMO1 (ab11672; Abcam, Cambridge, MA), anti-UBC9 (ab75854; Abcam, Cambridge, MA), anti-Bcl2 (ab692; Abcam, Cambridge, MA), anti-Bax (ab32503; Abcam, Cambridge, MA), anti-caspase3 (ab13847; Abcam, Cambridge, MA), anti-active caspase-3 antibody (ab2302; Abcam, Cambridge, MA), anti-Tom20 antibody (ab56783; Abcam, Cambridge, MA), anti-Tim23 antibody (ab116329; Abcam, Cambridge, MA), anti-Drp1 antibody (ab184247; Abcam, Cambridge, MA), anti-LC3B antibody (ab51520; Abcam, Cambridge, MA), anti-p62 antibody (ab56416; Abcam, Cambridge, MA), and anti-GAPDH antibody (ab128915; Abcam, Cambridge, MA). Primary antibodies were recovered and incubated with the secondary antibody for 1 h at room temperature. The membranes were visualized with the enhanced chemiluminescence reagents (Millipore, Boston, MA).

### 2.9. Immunoprecipitation

HL-1 cells were immunoprecipitated with antibodies to either SUMO1 or Drp1 using the Pierce™ Crosslink Magnetic IP/Co-IP Kit (88805, Thermo Fisher, Waltham, Massachusetts, USA) according to the manufacturer's instructions. The samples were then subjected to the standard Western blotting techniques and the membranes probed, respectively, with antibodies to Drp1 and SUMO1.

### 2.10. Experimental Protocols

Mouse hearts were subjected to 30 min of regional ischemia, followed by 2 h of reperfusion. The mice were treated with zinc (100 *μ*g/kg; Sigma, St. Louis, MO, USA) via the tail vein 5 min before reperfusion for 30 min. HL-1 cells were exposed to 1% O_2_ with Claycomb Medium for 24 h followed by 6 h of reoxygenation. Zn^2+^ chelator N,N,N′,N′-tetrakis-(2-pyridylmethyl) ethylenediamine (TPEN, 20 *μ*M) (Sigma-Aldrich, St. Louis, MO) was given 2 h after transfection with the vector or plasmid for 48 h.

### 2.11. Statistical Analysis

Data are expressed as mean ± SEM and were obtained from 5 to 10 separate experiments. Statistical significance was determined using the Student *t*-test or one-way ANOVA followed by Tukey's test. A value of *P* < 0.05 was considered as statistically significant.

## 3. Results

### 3.1. Effects of Zinc on SUMO Family Proteins under Normoxic and H/R Conditions

**Figure 1:**
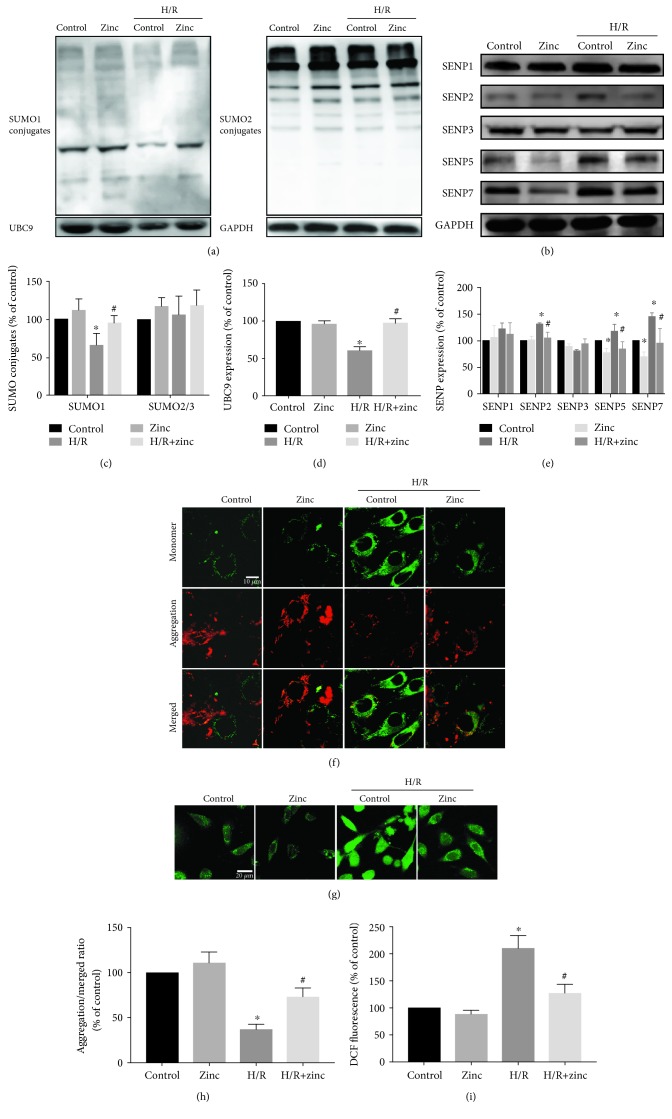
Effects of zinc on SUMO family members under physiological and H/R conditions. Compared with the control group, SUMO1 modification level of proteins and UBC9 in the H/R group decreased. The levels of SENP2, SENP5, and SENP7 decreased, while zinc (5 *μ*M) reversed the changes of SUMO1 and SENP2, SENP5, and SENP7 (a–e, *n* = 6). Compared with the H/R group, the aggregation/monomer ratio stained with JC-1 was reversed by zinc. Scale bar: 10 *μ*m (f, h; *n* = 10). Zinc inhibited H/R-induced increase of ROS detected by DCFH-DA. Scale bar: 20 *μ*m (g, i; *n* = 10). ^∗^*P* < 0.05 vs. control; ^#^*P* < 0.05 vs. H/R. H/R: hypoxia/reoxygenation.

Researches have reported that SUMOs play an important role in cerebral ischemia and cardiac ischemia. We have previously shown that zinc protects the heart against ischemia-reperfusion injury in isolated rat hearts and H9c2, so we investigate whether SUMO family proteins are involved in the protective effect of zinc on myocardial ischemia-reperfusion injury. As shown in Figures 1(a)–1(e) (*n* = 6), compared with the control group, H/R injury significantly decreased the SUMO1 conjugation of proteins. However, H/R injury did not affect the SUMO2/3 conjugation. Furthermore, the levels of SENP2, SENP5, and SENP7 decreased, whereas zinc reversed the effect of H/R on SUMO1, SENP2, SENP5, and SENP7. Under the normoxic conditions, zinc also downregulated SENP5 and SENP7 levels but did not have an effect on SUMO1 conjugation and SENP2 protein. Interestingly, the sole SUMO ligase UBC9 was also expressed at lower levels in the setting of ischemia/reperfusion, which was reversed by zinc. Together, these data suggest that SUMO1 conjugation and SENP2 levels are highly regulated by zinc in the setting of H/R injury but not under normoxic conditions. At the same time, H/R caused a loss of ΔΨm indicated by the decrease in the red (aggregate)/green (monomer) ratio and increased cellular ROS detected by DCF fluorescence intensity, which was reversed by zinc (Figures 1(f)–1(i), *n* = 10).

### 3.2. SUMO1 Contributes to the Protective Effect of Zinc on H/R Injury

**Figure 2:**
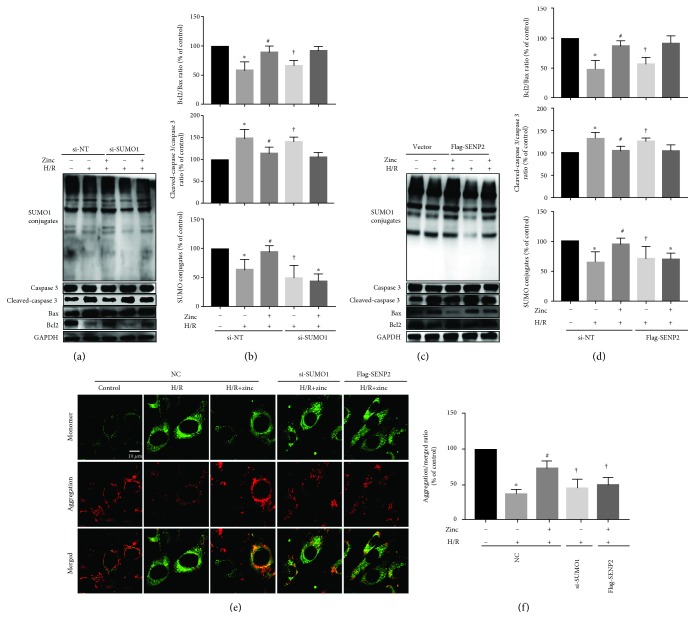
SUMO1 and SENP2 play an important role in the cardioprotective effect of zinc on H/R injury. Compared with the H/R group, zinc attenuated apoptosis by increasing the Bcl2/Bax ratio and decreasing the cleaved caspase3/caspase3 ratio, which were abolished by SUMO1 siRNA (a, b; *n* = 6) and overexpressed with SENP2 (c, d; *n* = 6). Compared with the H/R group, zinc inhibited the decrease of mitochondrial membrane potential detected by JC-1, which was abolished by SUMO1 siRNA (a, b; *n* = 6) and overexpressed with SENP2 (e, f; *n* = 7). Scale bar: 10 *μ*m. ^∗^*P* < 0.05 vs. control; ^#^*P* < 0.05 vs. H/R. ^†^*P* < 0.05 vs. H/R+zinc. H/R: hypoxia/reoxygenation; NC: negative control.

To examine if zinc protects cardiac cells from H/R injury via SUMO1, we determined the effect on the cleaved caspase3/caspase3 ratio, the Bcl2/Bax ratio, and the ratio of the JC-1 aggregation/monomer. As shown in Figures 2(a)–2(d) (*n* = 6), compared with the H/R group, zinc attenuated myocardial apoptosis by reducing the cleaved caspase3/caspase3 ratio but increasing the Bcl2/Bax ratio, which were reversed by SUMO1 siRNA and overexpressing SENP2. Similarly, compared with the H/R group, zinc increased the JC-1 ratio at reoxygenation, which was again abrogated by SUMO1 siRNA and overexpressing SENP2 (Figures 2(e) and 2(f), *n* = 7). These data suggest that SUMO1 contributes to the protective effect of zinc on H/R injury.

### 3.3. Zinc Protects the Heart against I/R Injury through SUMO1 in Isolated Mouse

**Figure 3:**
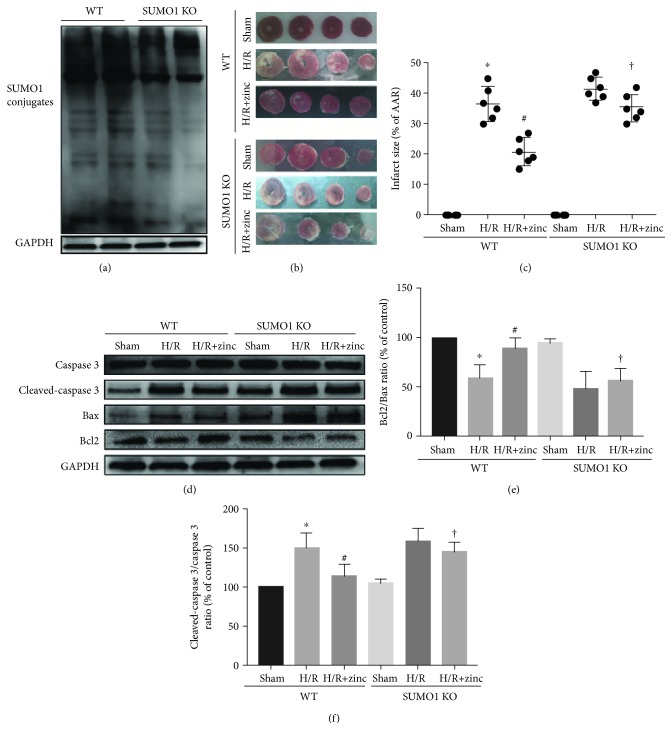
Effect of SUMO1 on zinc preconditioning-mediated myocardial injury reduction at the end of reperfusion in a Langendorff local I/R model. Compared to WT mice, the level of SUMOylation of proteins in the myocardium of SUMO1 KO mice decreased (a, *n* = 5). SUMO1 KO canceled the effect of zinc on attenuating myocardial infarct size (b, c; *n* = 6). Compared with the I/R group, zinc attenuated apoptosis by increasing the Bcl2/Bax ratio (d, e; *n* = 5) and decreasing the cleaved caspase3/caspase3 ratio (d, f; *n* = 5), which were abolished by SUMO1 KO. ^∗^*P* < 0.05 vs. control; ^#^*P* < 0.05 vs. I/R. ^†^*P* < 0.05 vs. I/R+zinc. AAR indicates area at risk; I/R: ischemia/reperfusion; SUMO1 KO: SUMO1 knockout.

To probe if elevated SUMO1 conjugation has an effect on the protective role of zinc, the SUMO1 knockout mice were subjected to 30 min regional ischemia followed by 2 h reperfusion. As shown in Figure 3(a) (*n* = 5), we observed variable decrease in the SUMO1 conjugations in the SUMO1 KO mice. The effects of zinc on IS and apoptosis were assessed in the WT and SUMO1 KO mice hearts. Compared to I/R, zinc reduced IS, and this effect of zinc was abolished by SUMO1 knockout (Figures 3(b) and 3(c), *n* = 6). In addition, zinc attenuated myocardial apoptosis by reducing the cleaved caspase3 ratio but increasing the Bcl2/Bax ratio, which were reversed by SUMO1 knockout (Figures 3(d)–3(f), *n* = 5).

### 3.4. Zinc Regulates the SUMOylation of Drp1

**Figure 4:**
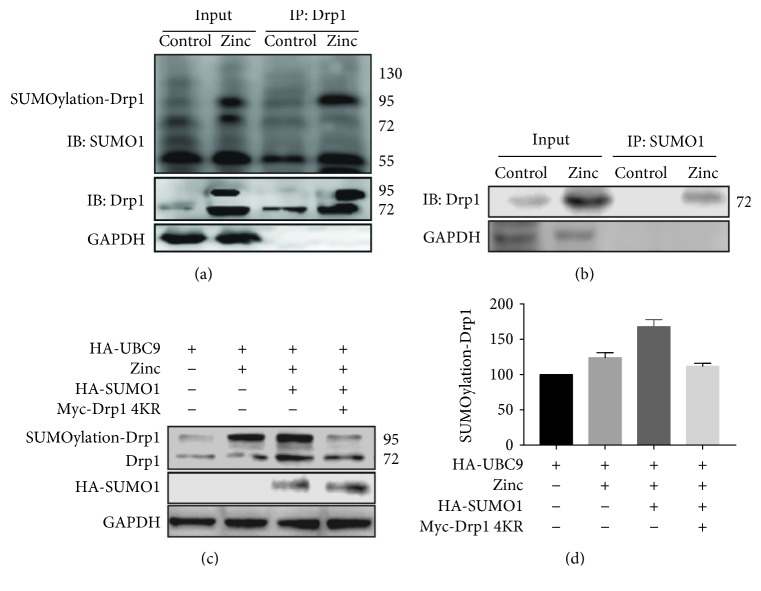
SUMOylation of Drp1 induced by zinc. Immunoprecipitation-immunoblot analyses examined the conjugation between Drp1 and SUMO1. Compared with the control group, zinc increased Drp1 SUMOylation (a, b; *n* = 5). Mutating four accepter lysines to arginines (Drp1 4KR) in cultured cardiomyocytes reduced zinc-induced Drp1 SUMOylation (c, d; *n* = 5). ^∗^*P* < 0.05 vs. zinc; ^#^*P* < 0.05 vs. zinc+HA-SUMO1.

Drp1 is a substrate of SUMO modification, so we test whether the protective role of zinc on I/R injury is dependent on Drp1 SUMOylation. Consistent with this, endogenous Drp1 interacts with SUMO1 in HL-1 cells and zinc treatment enhanced its interaction with SUMO1 (Figures 4(a) and 4(b), *n* = 5). To fully confirm the critical role of zinc on Dpr1 SUMO1-ylation, we made a non-SUMOylation Drp1 by mutating the four accepter lysines to arginines (Drp1 4KR). Compared to the zinc group, the SUMO1, UBC9, and Drp1 cotransfection increased the Drp1 SUMO1-ylation, which was abrogated by Drp1 4KR (Figures 4(c) and 4(d), *n* = 5). These data demonstrated that zinc regulates the Drp1 SUMO1-ylation.

### 3.5. Zinc Increased Mitophagy through SUMOylation of Drp1

**Figure 5:**
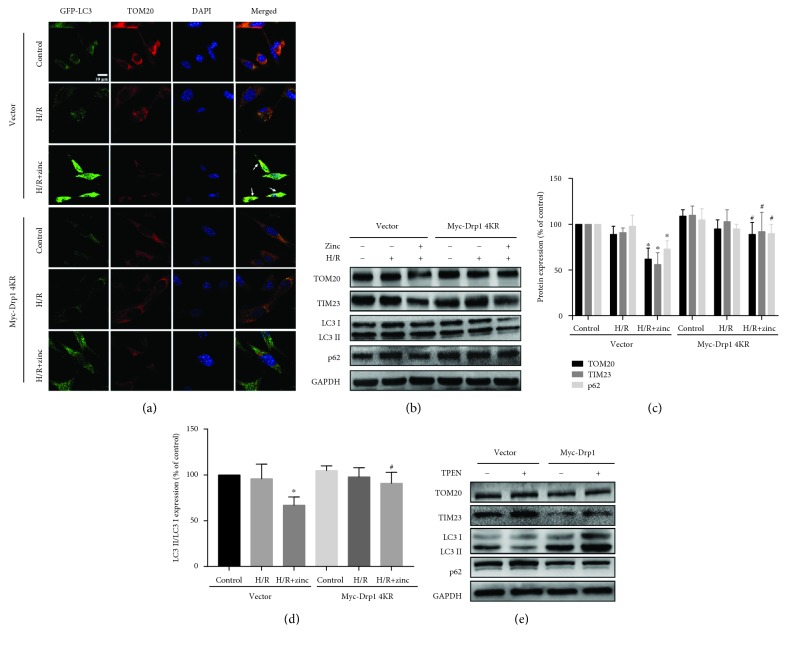
The role for Drp1 SUMO1-ylation in zinc-induced mitophagy. Confocal images of GFP-LC3 (green) and TOM20 (red). Increased LC3 was colocalized with TOM20 (a, *n* = 7). Effects of ZnCl_2_ on TOM20, TIM23, p62, and LC3 levels were inhibited by Myc-Drp1 4KR and Myc-Drp1 (b–e; *n* = 5). ^∗^*P* < 0.05 vs. H/R (Vector); ^#^*P* < 0.05 vs. H/R+ZnCl_2_ (Vector).

Previous reports suggested that Drp1 is downstream of the PINK1 signaling pathway. We have previously found that zinc induced autophagy via the PINK1 pathway; therefore, we next investigated whether SUMOylation of Drp1 induced by zinc could regulate mitophagy. As shown in Figure 5(a) (*n* = 7), compared to the H/R group, zinc increased the colocalization of GFP-LC3 and TOM20, which was reversed by Drp1 4KR, indicating that zinc might induce mitophagy through Drp1 SUMOylation. Similar findings were obtained in further studies using Western blotting; compared to the H/R group, zinc significantly increased the ratio of LC3-II/I but significantly downregulated levels of p62 and mitochondrial marker proteins TOM20 and TIM23 (Figures 5(b)–5(d), *n* = 5), which was reversed by Drp1 4KR. Further experiments showed that the selective zinc chelator TPEN suppressed the increase in LC3-II/I and decrease in TOM20 and TIM23 induced by Drp1 overexpression (Figure 5(e), *n* = 5).

### 3.6. SUMOylation of Drp1 Contributes to the Protective Effect of Zinc on H/R Injury

**Figure 6:**
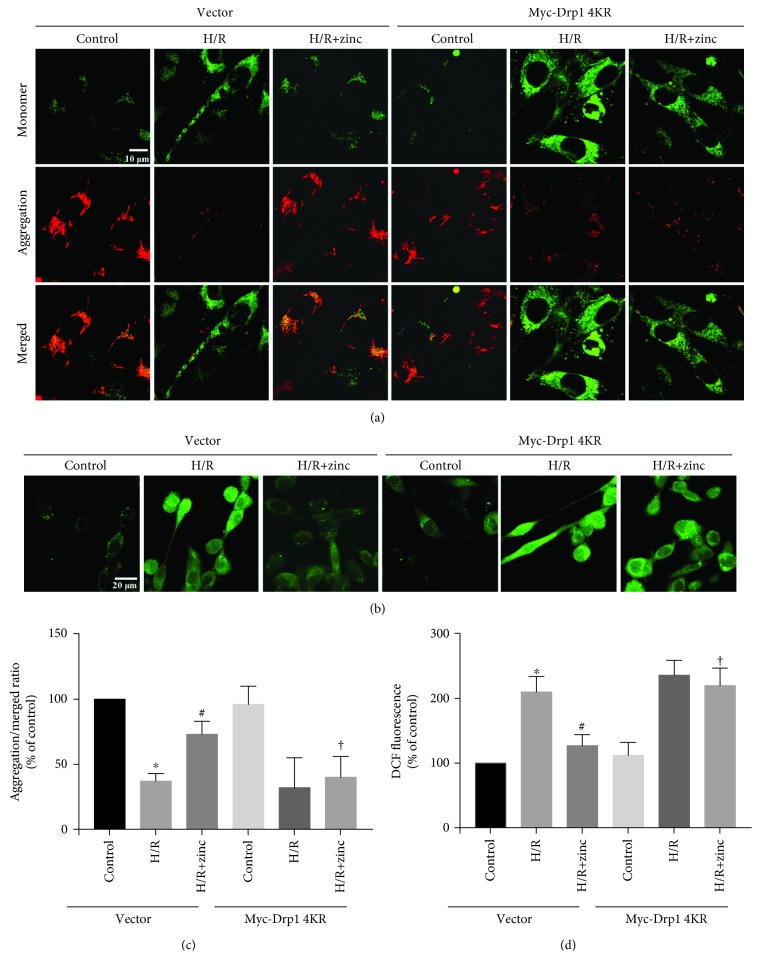
Effect of Myc-Drp1 4KR on zinc-mediated mitochondria protection in H/R-induced HL-1 cells. Compared with the H/R group, the aggregation/monomer ratio stained with JC-1 was reversed by zinc, which was abolished by Myc-Drp1 4KR (a, c; *n* = 5). Zinc inhibited H/R-induced increase of ROS, which was abolished by Myc-Drp1 4KR (b, d; *n* = 5). ^∗^*P* < 0.05 vs. H/R (Vector); ^#^*P* < 0.05 vs. H/R+ZnCl_2_ (Vector).

To test whether zinc prevents H/R injury through Drp1 SUMOylation, we determined the change of ΔΨm by detecting the ratio of the JC-1 aggregate/monomer. Compared to the H/R group, zinc increased the JC-1 ratio, which was suppressed by Drp1 4KR (Figures 6(a) and 6(c), *n* = 5). The DCF analysis showed that, compared to the H/R group, zinc decreased the DCF fluorescence intensity, which could be also abrogated by Drp1 4KR (Figures 6(b) and 6(d), *n* = 5).

## 4. Discussion

In this study, we have confirmed that SUMOylation of Drp1 plays an important role in the protective effect of zinc on H/R injury. When the heart suffers from I/R, SUMO1 conjugates are inactivated, zinc induced mitophagy via increasing Drp1 SUMO1-ylation. Mitophagy induced by Drp1 SUMOylation cleared damaged mitochondria, controlled mitochondrial quality, and prevented ROS generation, which improves myocardial function and reduces myocardial I/R damage.

Zinc is discovered in almost all biological tissues as one of the essential trace elements for the physiological and catalytic functions of the body [21]. Previous studies have reported that zinc is involved in myocardial differentiation and regeneration, arrhythmia, cardiac ischemia and reperfusion, and cardiac transplantation recovery [22]. Zinc given at reperfusion could reduce infarct size indirectly through signal factors, or directly as a signal molecule [23, 24]. Zinc is involved in cardioprotection indirectly through changing kinase activity, such as PI3K/Akt, ERK, glycogen synthase kinase 3*β* (GSK-3*β*), or by regulating the second messenger. For example, zinc alters intracellular second messenger cAMP activity by stimulating the activity of cyclic nucleotide phosphodiesterases (PDEs) [23, 25–27]. In addition, zinc is tightly linked to the cardioprotective effect through directly acting as intracellular signaling molecules [24]. In this context, we firstly reported that zinc increased the proteins of SUMOylation and decreased the levels of SENP2, SENP5, and SENP7. What is more, zinc given at reperfusion could protect mitochondria by increasing SUMO1 and decreasing the level of SENP2. SUMO1 knockout in mice heart significantly reversed the cardioprotective effect of zinc in the setting of H/R.

Although the global SUMO1 conjugations were significantly decreased at reperfusion, it is noteworthy to identify the target protein of SUMOylation modification. Mitochondria is the main source of superoxide generation under pathophysiological status in cardiomyocytes and mitochondria cleavage and fusion homeostasis plays an important role in I/R injury. Drp1 is a key protein necessary for mitochondrial division [28]. It has been reported that Drp1 SUMO2/3-ylation plays a cytoprotective role in the brain OGD model. SENP3 specifically cleaves SUMO2/3 from Drp1 and reduces apoptosis [29]. In contrast to the apoptotic effects reported for SUMO1 conjugation, Prudent et al. [30] reported that Drp1 SUMO1-ylation induced mitochondria division by recruiting Drp1 to mitochondria, leading to cell apoptosis. SENP5 overexpression mediated deSUMOylation of Drp1 and rescues Drp1 SUMO1-ylation-induced mitochondrial fragmentation. Consistent with this model, Jiang et al. discovered that Drp1 SUMO1-ylation increased the mitochondrial division, leading to neurodegeneration. In this model, SENP2 has an impact on the deSUMOylation of Drp1 [20]. Our data indicated that zinc has an adaptive protective response to I/R injury. Drp1 and its specific SENP may play different roles, which depend on the state of the cells and the animal models. Future research will be warranted to resolve these issues.

Drp1 was an important factor regulating mitochondrial division in cardiovascular disease [31]. Joshi et al. [31] have shown that endogenous Drp1 can bind to Bcl-1/Bcl-xL under physiological and I/R stress conditions. Bcl-1/Bcl-xL is an endogenous inhibitor of the autophagy-inducing factor Beclin1. Consistent with this, Drp1 knockout suppressed autophagy by increasing the conjugation of Beclin1 and Bcl-1/Bcl-xL, which resulted in mitochondrial dysfunction and cardiomyocyte apoptosis. Similarly, our finding showed that zinc induced mitophagy by increasing SUMOylation of Drp1, which led to damaged mitochondrial clearance and prevention of ROS generation in the setting of cardiac I/R. Although it is the first time to report such a protective mechanism of zinc, a mass of questions remain to be future researched.

In summary, this study demonstrated that SUMOylation of Drp1 played an essential role in zinc-induced cardioprotection against I/R injury. SUMOylation of Drp1 caused by zinc increased mitophagy at reperfusion as a result of prevention of ROS and myocardial injury. SUMO1, as a posttranslational modification factor, mediates the action of zinc through Dpr1, which provides a new insight into the mechanisms underlying cardioprotection of zinc. We believe that our findings certainly warrant further investigations for application in the clinical setting.

## Data Availability

All the data used to support the findings of this study are available from the corresponding authors upon request.
